# Inhibition of HMGB1 Promotes Osseointegration under Hyperglycemic Condition through Improvement of BMSC Dysfunction

**DOI:** 10.1155/2019/1703709

**Published:** 2019-12-19

**Authors:** Beilei Liu, Xueqi Gan, Yuwei Zhao, Hongdou Yu, Jing Gao, Haiyang Yu

**Affiliations:** State Key Laboratory of Oral Disease, National Clinical Research Center for Oral Diseases, West China Hospital of Stomatology, Sichuan University, Chengdu 610041, China

## Abstract

High mobility group box 1 (HMGB1) participates actively in oxidative stress damage and the latter relates closely to diabetic complications, including poor implant osseointegration. This article is aimed at investigating the effects of HMGB1 on dysfunction of bone marrow stromal cells (BMSCs) and impaired osseointegration under diabetic environment. *In vitro*, BMSCs were treated with normal glucose (NG), high glucose (HG), and HG+glycyrrhizin (HMGB1 inhibitor, HG+GL). Cell proliferation, osteogenic behaviors, and oxidative stress were determined. *In vivo*, 8-week-old Sprague-Dawley rats were categorized to control, streptozotocin-induced diabetic, and diabetic-GL groups. Rats received GL (50 mg/kg, i.p.) or vehicle treatment daily after titanium implants were planted into the tibiae. After 4 and 8 weeks, plasma lipoperoxide detection, *μ*CT analysis, and histomorphometric evaluation were conducted. By these approaches, we demonstrated that inhibiting HMGB1 by GL significantly attenuated HG-induced upregulation of HMGB1, HMGB1 ligand receptor for advanced glycation end products (RAGE) and their interaction, relieved oxidative stress, and reversed the downregulation of osteogenic markers, resulting in improved osteogenic differentiation. In diabetic rats, GL administration suppressed the upregulation of HMGB1, attenuated the lipoperoxide, and ameliorated the impaired trabecular structure and osseointegration. Taken together, inhibiting HMGB1 can be an effective approach to relieve BMSC dysfunction and enhance osseointegration under diabetic environment.

## 1. Introduction

Diabetes mellitus has become a global challenge with its prevalence rate rising at an alarming rate. There is a high prevalence of periodontal disease with resultant tooth loss in diabetic patients [1]. Implant-supported denture is considered to be a favorable treatment to restore lost teeth and rehabilitate occlusion, as long as a reliable osseointegration is achieved between the bone and implant. Although dental implant has been described as a safe therapy with a high success rate, diabetes remains a relative contraindication for implant treatment [2] due to its association with oxidative stress [3], delayed wound healing [4], and increased proinflammatory cytokines during osseous healing [5]. Implant in diabetic animals exhibited attenuated bone regeneration capacity and compromised osseointegration [6]. However, the underlying mechanisms remain elusive.

High mobility group box 1 (HMGB1) is known as a nonhistone nuclear protein regulating gene expression. In the late 1990s, HMGB1 was rediscovered as an endogenous danger signal molecule. It can provoke inflammatory responses when released to extracellular milieu under conditions of mechanical trauma, oxidative stress, acute and chronic inflammatory such as diabetes mellitus, and periodontal disease [7, 8]. According to recent researches, plasma HMGB1 levels significantly increased in diabetic patients and HMGB1 was involved in the pathological development of diabetes and related complications by regulating cellular response to oxidative stress and its proinflammatory effect [9, 10].

HMGB1 is a bone-active cytokine. According to Charoonpatrapong [11], osteoblast-lineage cells express HMGB1. Tan found that hypoxia enhanced the expression of HMGB1 in rat bone marrow stromal cells (BMSCs), inhibiting cellular proliferation and apoptosis behaviors [12]. HMGB1 can function as cytokine and chemokine activator when associated with its receptors and activate the downstream signaling pathway. HMGB1 receptors include receptor for advanced glycation end products (RAGE) [13], toll-like receptors (TLRs, e.g., TLR2, TLR4) [14]. RAGE activation has been implicated in diabetes, cancer, and Alzheimer's disease. Moreover, RAGE is a part of the underlying molecular link among diabetes, osteoporosis, and reactive oxygen species (ROS) overproduction cascade [15, 16]. While HMGB1 interactions with TLRs may also be important, the HMGB1-RAGE interaction plays a more important part in the context of diabetes and periodontal diseases [17].

Therefore, this study is aimed at evaluating the implication of HMGB1 in the pathological process of diabetic BMSC dysfunction, osseointegration impairment, and the underlying mechanisms, with a final goal of providing novel strategies to achieve improved osseointegration in diabetic patients.

## 2. Materials and Methods

### 2.1. BMSC Isolation and Treatment

Rat BMSCs were isolated from the tibiae and femurs of 2-week-old male Sprague-Dawley (SD) rats. Briefly, the rats were sacrificed by cervical dislocation and soaked in 75% ethanol for 5 min. Then, the femurs and tibias were isolated. Disposable aseptic syringes were used to repeatedly wash the bone marrow cavity to collect cells in petri dishes with alpha-minimal essential medium (*α*-MEM; Gibco, USA) containing 10% fetal bovine serum (Gibco, Australia) and antibiotics. The obtained cell suspension was then transferred to plastic culture flasks for incubation. BMSCs of the third passage were used for experiments.

Glycyrrhizin (GL) is a specific HMGB1 inhibitor [18]. To determine the role of HMGB1 in high glucose-induced BMSC dysfunction, cells were cultivated in osteogenic medium containing physiological normal glucose (NG; 5.5 mM), high glucose (HG; 35 mM), or high glucose with glycyrrhizin (HG+GL; 35 mM glucose+10 *μ*M GL). The osteogenic medium contained 10 mM *β*-glycerophosphate disodium, 50 *μ*g/ml ascorbic acid, and 100 nM dexamethasone. For GL treatment, glycyrrhizin (#50531, Sigma-Aldrich, USA) powder was dissolved in DMEM medium at a stock concentration of 10 mM and sterilized by filtration (0.22 mm, Millipore), then stored at 4°C. Before every experiment, an aliquot of GL was thawed and diluted to a working concentration of 10 *μ*M prior to use.

### 2.2. Cell Viability

Cell viability evaluation was performed by use of Cell Counting Kit-8 (Dojindo, Japan). BMSCs were seeded in a 96-well plate at a density of 5 × 10^3^ cells/well. After attachment, the medium was replaced by NG, HG, and HG+GL medium. CCK-8 assay was conducted after 3, 7, and 14 days. All medium was replaced by 100 *μ*L *α*-MEM containing 10% CCK-8. After incubation at 37°C for 1 h, BMSC viability was determined using a microplate reader (Thermo Scientific, Varioskan Flash, USA) at 450 nm.

### 2.3. Cell Differentiation

To evaluate the osteogenic differentiation function of BMSCs, alkaline phosphatase (ALP) staining was performed on the 3rd, 7th, and 14th day and alizarin red S staining was performed on the 21st day. After fixed in 4% paraformaldehyde for 15 min and washed with phosphate-buffered saline (PBS), the BMSCs were stained with alkaline phosphatase assay kit (Nanjing Jiancheng, China) for 30 min or alizarin red S (1%, Sigma, pH = 4.2) for 15 min at room temperature. The incorporated alkaline phosphatase and calcium were visualized under a microscope (Olympus, Japan). Then, the mineralized nodules were eluted with 10% cetylpyridinium chloride and quantified using a microplate reader at 570 nm.

### 2.4. Intracellular ROS Level

5 × 10^4^ cells/well were seeded in 12-well plates and incubated with different media for 72 h prior to assessment. 2,7-Dichlorofluorescein diacetate (DCFA; Sigma) staining was conducted to detect intracellular ROS content. Fluorescent images were obtained using a microscope (Olympus, Japan). ImagePro Plus (Media Cybernetics, USA) was used to measure fluorescent signal density after image acquisition.

### 2.5. Quantitative Real-Time-PCR

1 × 10^5^ cells/well were seeded in 6-well plates. Total RNA was extracted by the use of a RNA Extraction Kit (TAKARA, Japan). The concentration and purity of extracted RNA were evaluated by NanoDrop (Thermo Scientific, USA). After cDNA was synthesized, real-time quantitative PCR was conducted using SYBR® Premix *Ex Taq*™ II (TAKARA, Japan) on an ABI QuantStudio 3 PCR System (Applied Biosystems, USA). The primer sequences for HMGB1, RAGE, heme oxygenase-1, Runx2, ALP, OCN, OPG, RANKL, and *β*-actin are listed in Table 1. The expressions of detected genes were figured out by 2^−*∆∆*CT^ method.

### 2.6. Western Blot and Coimmunoprecipitation

Rat bone tissue or BMSCs pretreated with different media for 14 days were homogenized in lysis buffer (140 mM NaCl, 1 mM EDTA, 10% glycerol,1% NP40, 20 mM Tris-HCl, pH 7.5, containing protease-inhibitor, 1 mM PMSF), centrifuged, and used for western blot. 20 *μ*g lysates were loaded to 10% SDS-PAGE and transferred to PVDF membranes. After blocked in 5% nonfat milk for 1 h, the membranes were incubated with different primary antibodies at 4°C overnight, namely anti-HMGB-1 (1 : 1000, #6893S, CST), anti-RAGE (1 : 1000, #ab3611, Abcam), anti-HO-1 (1 : 1000, #ab13248, Abcam), anti-OPG (1 : 1000, #ab73400, Abcam), anti-RANKL (1 : 1000, #ab9957, Abcam), and anti-*β*-actin (1 : 8000, #ab6276, Abcam). After a wash step, the membranes were incubated with secondary anti-rabbit IgG antibody (1 : 10000, #ab6721, Abcam) or anti-mouse IgG antibody (1 : 5000, #ab6789, Abcam). Immunoreactive protein bands were visualized using a chemiluminescence machine (Bio-Rad, USA). Relative optical density of band was detected by NIH ImageJ software and analyzed relative to *β*-actin levels.

To investigate the HMGB1-RAGE binding, immunoprecipitation experiments were carried out by using anti-HMGB1 antibody, and detecting of the HMGB1-associated complex binding to RAGE afterwards. Protein samples were added to anti-HMGB1 antibody (10 *μ*g/mL, #ab118256, Abcam) and rotated at 4°C overnight. Then, prewashed protein A/G plus agarose beads were added. After incubation, immunoprecipitates were washed with lysis buffer, resuspended in 50 *μ*L Laemmli sample buffer, analyzed by gel electrophoresis, and then transferred to PVDF membranes. Immunoblotting with anti-RAGE antibody (10 *μ*g/mL, #ab181293, Abcam) was conducted afterwards.

### 2.7. Diabetes Mellitus Molding

Eight-week-old specific-pathogen-free (SPF) male SD rats were provided by the Experimental Animal Center of Sichuan University. The animals were kept in plastic cages in a climate-controlled house and fed with standard laboratory diet and water *ad libitum*. After an acclimatization period of 1 week, the rats were randomly categorized to control, diabetic, or diabetic-GL groups. After an overnight fast, rats in the diabetic and diabetic-GL groups received intraperitoneal injection of streptozotocin (60 mg/kg, Sigma, USA). Rats in the control group received injection of vehicle. The nonfasting blood glucose level (BGL) was recorded weekly using an electronic glucometer (Accu-Check Performa, Roche Diagnostics, USA). BGL > 16.7 mmol/L was regarded as a success of diabetic molding. Those rats experiencing failed molding or death were rejected from this experiment.

### 2.8. Implant Surgery and Glycyrrhizin Treatment

**Figure 1:**
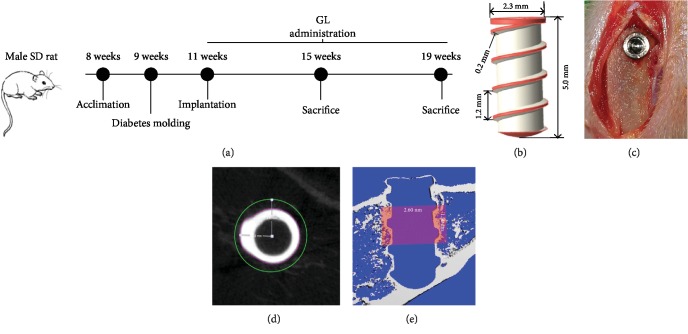
The design of *in vivo* study. (a) Time schedule of *in vivo* study. (b) Design graph of the screw-shaped titanium implant. (c) Implant in the tibia. (d, e) VOI of the peri-implant trabecular for *μ*CT evaluation. VOI was defined as a hollow cylinder from 1.0 mm below the tibia cortex to 150 slices towards the bone marrow, extending with a radius of 350 *μ*m from the implant body surface, which is 150 *μ*m from the threads, between the green and purple circles in the graph.

The titanium implants (Figure 1(b), *ϕ*2.3 × 5.0 mm) were designed and manufactured by Puchuan Biomaterials Corporation (Chengdu, China) according to the requirements of researchers. To induce anesthesia, all rats were injected with a combination of ketamine (0.08 mg/kg) and xylazine (0.04 mg/kg). Further anesthesia was conducted by a local injection of lidocaine (2%, with adrenalin 1 : 100,000) to assure analgesia in surgical site. A full-thickness incision was operated on the right tibia below the knee joint. Tissues were reflected to expose the bone. The implant cavity was prepared by a dental hand piece with a pilot drill under the irrigation of precooled sterilized physiological saline. After bone chips were cleaned, the implant was screwed into the holes, shown in Figure 1(c). The soft tissues were sutured in situ. A single intramuscular injection of penicillin (100,000 IU) was administered immediately after surgery and in the following 3 days to prevent infection.

HMGB1 knockout mice were generated in earlier study but died within 24 hours after birth due to insufficient glucocorticoid receptor and hypoglycemia [19]. Due to the restrained application of HMGB1 knockout animals, GL was used to inhibit HMGB1 for the *in vivo* study. Rats in the diabetic-GL group received administration of GL (50 mg/kg) immediately after surgery and daily thereafter through intraperitoneal injection. Animals in the control and diabetic groups received vehicle injection of normal saline.

### 2.9. Determination of HMGB1, SOD, GSH-Px, and MDA Levels

After a healing period of 4 weeks and 8 weeks, the rats were sacrificed by intraperitoneal injection of pentobarbital in overdose. Plasma samples of each group were collected and stored at -80°C. ELISA kits (ARG81310, Arigobio) were used to quantify plasma levels of HMGB1 according to the manufacturer's instructions. Spectrophotometer method was used to evaluate malondialdehyde (MDA), superoxide dismutase (SOD), and glutathione peroxidase (GSH-PX) levels.

### 2.10. *μ*CT Analysis

Tibiae containing implants were separated and fixed in 10% formaldehyde for 72 h. The metaphysis of the tibia containing implants were scanned by *μ*CT 50 (Scanco Medical, Switzerland) with a voxel size of 10 *μ*m, energy setting of 90 kV, 114 mA, and an integration time of 500 ms. Raw images were reconstructed and analyzed by SCANCO Medical Evaluation and Visualizer software. The volume of interest (VOI) was defined as a hollow cylinder from 1.0 mm below the tibia cortex to 150 slices towards the bone marrow, extending as a radius of 350 *μ*m from the implant body surface, that is 150 *μ*m from the threads (Figures 1(d) and 1(e)). Bone volume fraction (BV/TV), mean trabecular number (Tb.N), mean trabecular thickness (Tb.Th), and mean trabecular separation (Tb.Sp) were calculated within the VOI. The ratio between bone voxels and total voxels in direct contact to implant surface was assessed and named as bone-to-implant contact (BIC) rate.

### 2.11. Histomorphometry and Immunohistochemistry (IHC)

After *μ*CT analysis, samples without decalcification were dehydrated in a graded ascending series of ethanol (70–100%), infiltrated and embedded in methyl methacrylate. The specimens were sawn along the implant axis by a Leica diamond saw (SP1600, Germany). All sections were ground and polished to a thickness of 80 *μ*m and stained with Masson trichrome staining. Histomorphometric analysis was performed on all section images acquired from Nikon ECLIPSE 80i microscope (Germany). Postacquisition analysis was performed using ImageJ software to calculate the BIC rate, defined as the linear percentage of the bone in direct contact to the implant and the total interface of the implant in the tibia.

After *μ*CT analysis, samples after decalcification were dehydrated and embedded in paraffin. Transverse tissue sections along the longitude of the tibia were subjected to IHC staining. Briefly, all sections were deplasticized, rehydrated, and blocked for endogenous peroxidases by treatment with hydrogen peroxide. After antigen retrieval, all sections were incubated in serum-free protein block solution for 30 min to prevent nonspecific binding. Afterwards, sections were incubated with primary antibodies against HMGB1, RAGE, HO-1, Runx2, OCN, OPG, and RANKL at 4°C overnight. Each primary antibody was used at a 1 : 200 dilution following the protocol. After incubation with secondary anti-rabbit or anti-mouse IgG antibodies, detection of primary antibodies was visualized using a DAB-horseradish peroxidase substrate system. Imaging of the slice was performed under a light microscope (Nikon Eclipse 600, Japan).

### 2.12. Statistical Methods

Data are presented as mean ± SD. Statistical analysis was performed using GraphPad Prism 7.0 software (GraphPad Software, USA). One-way ANOVA or two-way ANOVA and Tukey's multiple comparisons tests were adopted to determine the level of significance. Statistical significance was determined as *P* < 0.05.

## 3. Results

### 3.1. Glycyrrhizin Suppressed HMGB1 Upregulation in HG-Treated BMSCs

**Figure 2:**
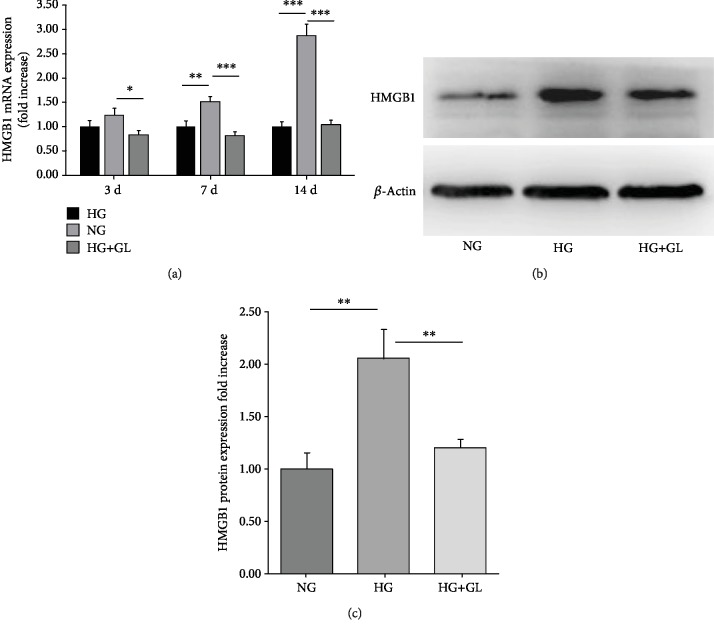
GL suppressed HMGB1 upregulation in HG-treated BMSCs. (a) Relative gene expression of HMGB1 at 3 d, 7 d, and 14 d in different groups. Representative band (b) and quantification (c) of protein expression of HMGB1 after treatment for 14 days. All data were normalized to *β*-actin. ^∗^*P* < 0.05, ^∗∗^*P* < 0.01; ^∗∗∗^*P* < 0.001; *n* = 3.

As shown in Figure 2(a), the markedly upregulated gene expression of HMGB1 was seen in the HG group. HMGB1 mRNA expression increased over time from 3 d to 14 d. At 14 d, HMGB1 mRNA expression reached a 2.88-fold increase in HG group while GL suppressed HMGB1 expression by 63.4%. Western blot analysis showed a further verification to mRNA evaluation, where HMGB1 protein expression reached a 2.05-fold increase in the HG group and GL treatment inhibited its expression to 1.20-fold (Figures 2(b) and 2(c)).

### 3.2. Inhibition of HMGB1 Relieved BMSC Dysfunction under HG Condition

**Figure 3:**
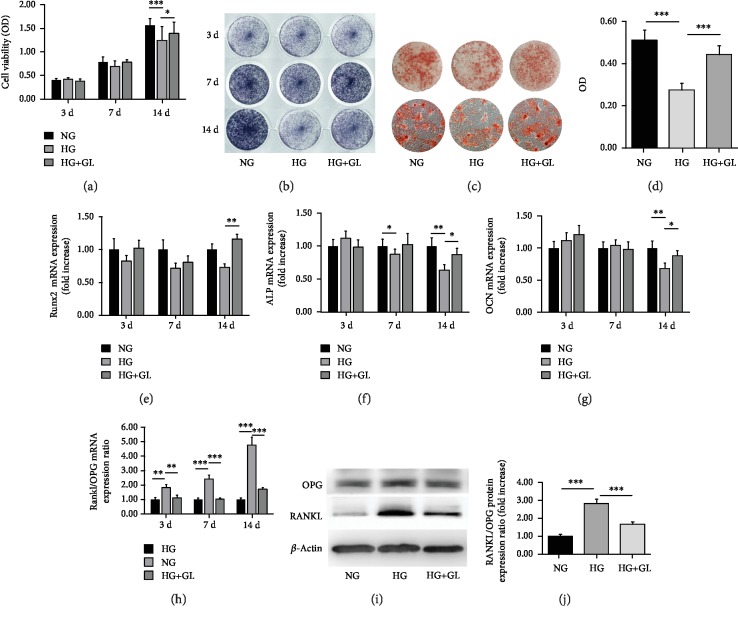
Inhibiting HMGB1 by GL relieved BMSC dysfunction under HG condition. (a) Cell viability evaluated by the CCK-8 assay at 3 d, 7 d, and 14 d, *n* = 6. (b) ALP staining of BMSCs after an osteogenic induction of 3 d, 7 d, and 14 d. (c) Alizarin red staining of BMSCs after an osteogenic induction of 21 days and deposited calcium stains red. (d) Quantification of mineralization nodules in different groups, *n* = 3. Relative expressions of Runx2 (e), ALP (f), OCN (g), and RANKL/OPG (h) detected at 3 d, 7 d, and 14 d by RT-qPCR analysis, *n* = 3. Representative band (i) and quantification (j) of protein levels of OPG and RANKL at 14 d, *n* = 3. All data were normalized to *β*-actin. ^∗^*P* < 0.05, ^∗∗^*P* < 0.01; ^∗∗∗^*P* < 0.001.

As shown in Figure 3(a), a significant decrease in cell viability was noted in HG group compared to NG group (*P* < 0.05) by day 14, while GL treatment rescued cell viability to a small extent (*P* < 0.05).

To evaluate osteogenic differentiation of BMSCs, ALP and alizarin red S staining were performed. ALP staining in Figure 3(b) shows that the expression of ALP decreased in the HG group. Similarly, mineralization capacity of the osteoblastic BMSCs was evaluated by alizarin red S staining (Figures 3(c) and 3(d)). In the NG group, many calcium nodules were found in BMSCs, exhibiting as large calcifying foci accompanied by smaller foci in development. In the HG group, the calcifying nodules decreased. Moreover, the nodules were smaller and poorly mineralized. To verify that, we measured the gene expression of osteogenic markers. The mRNA expression levels of ALP, Runx2, and OCN notably decreased in HG-treated BMSCs at 14 days (Figures 3(e), 3(f), and 3(g)). These results indicated that osteogenic differentiation of BMSCs was hampered in HG group. After inhibiting HMGB1 by GL, osteogenic gene expression increased; therefore, produced ALP levels and calcific nodules are significantly higher than the HG group. Moreover, we assessed gene expressions of OPG and RANKL, which act as osteoclastogenesis regulators and bone inflammatory makers. Although HG did not cause any significant change in the expression of OPG, it caused an impressive increase in RANKL mRNA and protein levels. Interestingly, inhibiting HMGB1 by GL significantly prevented this high glucose-induced RANKL upregulation (Figures 3(h), 3(i), and 3(j)).

### 3.3. ROS Accumulation in BMSC Dysfunction under HG Condition and the Involvement of HMGB1-RAGE Interaction

**Figure 4:**
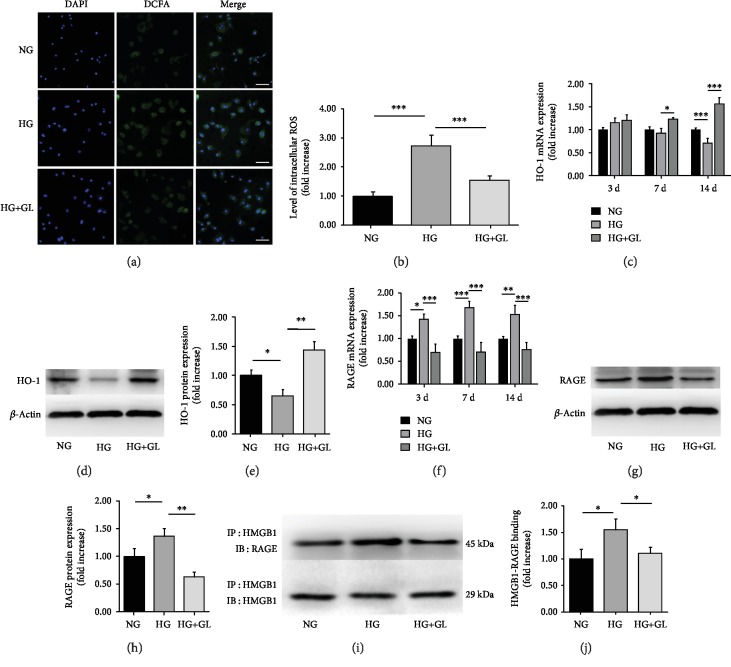
HMGB1-RAGE interaction is involved in ROS accumulation. Representative images (a) and quantification of fluorescence intensity (b) of DCFA staining after an incubation of 72 h. ROS stains are green while nuclei stain is blue. Scale bar = 200 *μ*m. Relative expression of mRNA (c) and protein level (d, e) of HO-1. Changes of mRNA (f) and protein level (g, h) of RAGE. All data were normalized to *β*-actin. HMGB1-RAGE binding activity (i, j) was determined by performing coimmunoprecipitation assay after incubation for 3 days. *n* = 3. ^∗^*P* < 0.05, ^∗∗^*P* < 0.01; ^∗∗∗^*P* < 0.001.

ROS are natural by-products produced during cellular metabolism. However, under pathologic conditions such as diabetes, ROS production and accumulation increase markedly and cause dramatic damages to cellular structures. In this experiment, ROS levels were measured in BMSCs to determine the effect of HMGB1 on cellular oxidative stress. As shown in Figures 4(a) and 4(b), significant accumulation of ROS was detected in HG group, as a stronger green fluorescence was detected compared to the NG group. In the HG+GL group, the accumulation of ROS notably receded. HO-1, an oxidative stress regulator, increased after short-term stimulation of HG but decreased following relative long-term of incubation (Figures 4(c), 4(d), and 4(e)). Inhibiting HMGB1 by GL improved HO-1 mRNA and protein expressions at 14 days, indicating a potential effect of alleviating oxidative stress in HG-treated BMSCs.

To investigate the role of HMGB1 employed in oxidative stress, HMGB1-RAGE interaction was evaluated. RAGE can function as a HMGB1 ligand and has been demonstrated to activate diverse signal transduction cascades including generation of ROS in diabetes [16, 20]. In HG-treated BMSCs, RAGE expression reached a 1.53-fold increase in mRNA and a 1.36-fold increase in protein (Figures 4(f), 4(g), and 4(h)). Moreover, as evidenced by coimmunoprecipitation assays (Figures 4(i) and 4(j)), notably increased HMGB1-RAGE binding was found in HG-treated BMSCs. While in the presence of glycyrrhizin, decreased binding of RAGE coprecipitated with HMGB1was observed.

### 3.4. HMGB1 Upregulation in Diabetic Rats and Its Relationship with Oxidative Stress

**Figure 5:**
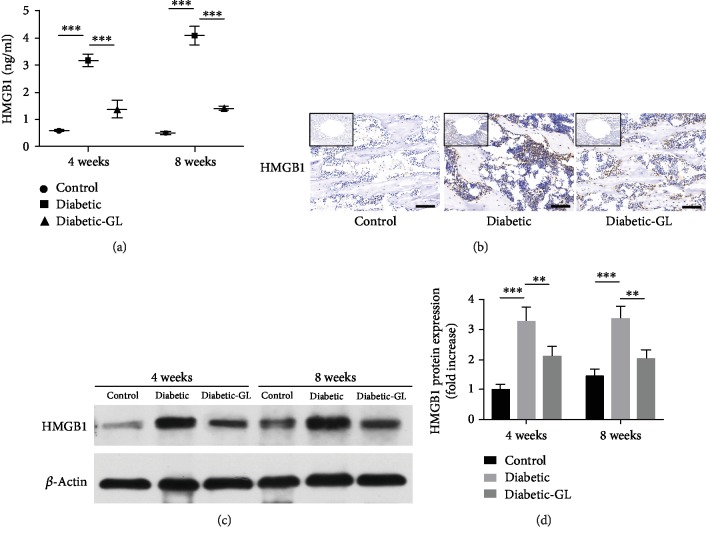
HMGB1 upregulated in diabetic rats. (a) Plasma concentration of HMGB1, *n* = 6. (b) Immunochemistry evaluation of HMGB1 in trabecular around the implant. Scale bar = 100 *μ*m. Representative band (c) and quantification (d) of HMGB1 protein levels of rat bone tissue, *n* = 3. ^∗^*P* < 0.05, ^∗∗^*P* < 0.01; ^∗∗∗^*P* < 0.001.

*In vivo* study design was descripted in the method part and illustrated in Figures 1(a)–1(e). As shown in Figure 5(a), plasma HMGB1 is approximately 0.60 ng/ml in the control group. While in diabetic group, plasma HMGB1 concentration increased to 3.17 ng/ml at 4 weeks and reached 4.09 ng/ml at 8 weeks. This high concentration of plasma HMGB1 in diabetic rats was markedly reduced after GL treatment (Figure 5(a)). IHC staining shows that HMGB1 was clearly upregulated in the bone tissue of diabetic rats compared to normal rats (Figure 5(b)). While under GL treatment, the overexpression of HMGB1 was downregulated. In addition, the expression of HMGB1 in the bone tissue of diabetic rats was significantly higher than that in the control group. As shown in Figures 5(c) and 5(d), HMGB1 protein expression reached 3.29-fold and 3.37-fold increases 4 and 8 weeks after implantation (*P* < 0.001), respectively. Daily intraperitoneal injection of HMGB1 inhibitor GL can significantly inhibit the expression of HMGB1 in the bone tissue of diabetic rats, which decreased to 2.10-fold and 2.03-fold in comparison to the control group after 4 and 8 weeks, respectively.

**Figure 6:**
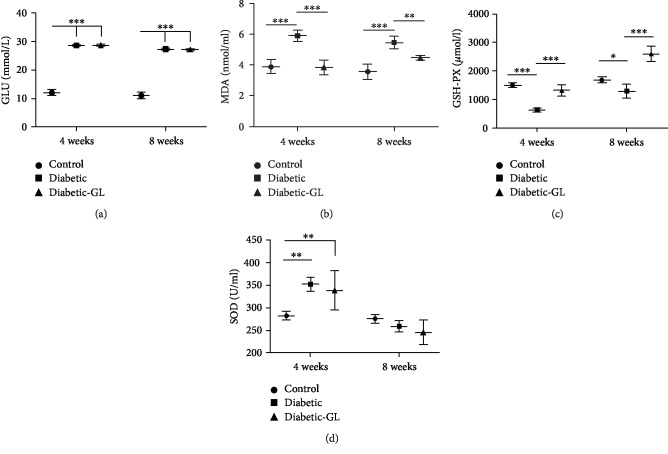
Inhibiting HMGB1 relieved oxidative stress in diabetic rats. Plasma concentration of glucose (a), MDA (b), GSH-PX (c), and SOD (d), *n* = 6. ^∗^*P* < 0.05, ^∗∗^*P* < 0.01; ^∗∗∗^*P* < 0.001.

In animal study, blood glucose levels of rats in diabetic group and diabetic-GL group ranged from 25.0 to 32.0 mmol/L and were maintained throughout the study (Figure 6(a)). Glycyrrhizin administration could not significantly relieve the hyperglycemia condition of diabetic rats. To examine the implication of HMGB1 on oxidative stress in diabetic rats, the levels of MDA, SOD, and GSH-PX were evaluated by spectrophotometry. MDA is formed during the decomposition of lipid peroxidation products. SOD and GSH-PX act as free radical scavengers that could prevent ROS generation [21]. MDA level significantly increased (Figure 6(b)) while GSH-PX level significantly decreased (Figure 6(c)) in plasma of diabetic rats compared to the control group (*P* < 0.05). Moreover, SOD activity increased in diabetic rats at the first 4 weeks but decreased significantly in the diabetic rats by 8 weeks (Figure 6(d)).

### 3.5. Inhibiting HMGB1 Rescued the Impaired Osseointegration in Diabetic Rats

**Figure 7:**
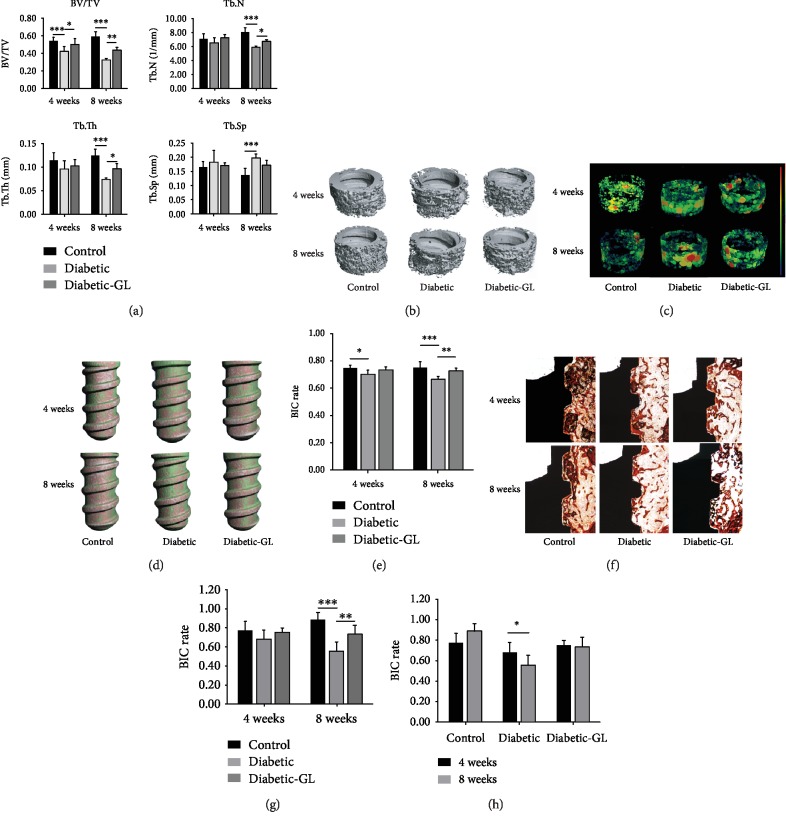
Inhibiting HMGB1 rescued impaired osseointegration in diabetic rats. (a) Quantification of BV/TV, Tb.N, Tb.Th, and Tb.Sp within the VOI. (b) Morphology of peri-implant trabeculae. Four weeks after implantation, the trabecular thickness and number were quite similar in three groups. After another healing period of 4 weeks, however, the trabeculae became sparser and thinner among rats in the diabetic group. While in the control group, the trabeculae got denser and well organized. In the diabetic-GL group, the reduction in trabecular number caused by diabetes was relieved, but not eliminated. (c) Morphology of peri-implant trabecular separation. In the diabetic group, the intertrabecular space is filled with macro yellow-to-red bubbles, indicating large space and big separation. While in the control group, the space is filled with micro green-to-blue bubbles, representing thin space and small separation. (d) Representative images of BIC by *μ*CT evaluation. The pink area represents bone in direct contact with the implant. (e) Quantification of BIC by *μ*CT evaluation. Representative images (f) and quantification (g, h) of BIC under histomorphometry, showing the newly formed bone tissue adjacent to the implant surface (Masson trichrome staining). *n* = 6. ^∗^*P* < 0.05, ^∗∗^*P* < 0.01; ^∗∗∗^*P* < 0.001.

To determine the effects of HMGB1 on trabecular morphology and BIC under diabetic condition, *μ*CT evaluation, histomorphometry, and IHC staining were performed. From 4 weeks to 8 weeks, mean BV/TV, Tb.N, and Tb.Th in the control group increased and Tb.Sp decreased (Figure 7(a)). In the diabetic group, however, mean BV/TV decreased from 0.42 to 0.33, resulting from a decrease of mean Tb.N and Tb.Th. Likewise, BV/TV, Tb.N and Tb.Th in the diabetic-GL group also decreased, but to a more moderate extent. After a healing period of 4 weeks, significant reduction of the peri-implant trabecular BV/TV was observed in diabetic group, in comparison with the control group. When it came to 8 weeks, this significance enlarged. Compared to that at 4 weeks, quantitative evaluation (Figure 7(a)) showed that BV/TV, Tb.N, and Tb.Th of rats in the control group increased, while those in diabetic group dropped; as to the diabetic-GL group, BV/TV, Tb.N, and Tb.Th remained nearly the same as they were at 4 weeks, though slight reduction was observed. In the diabetic group, bone volume around the implant was dramatically less than that in control group (*P* < 0.05), and GL treatment significantly increased the BV/TV by 10.8% and Tb.N by 3.3% (*P* < 0.05). As to Tb.Th, a similar effect was found but to a more moderate extent (*P* > 0.05). 3D stereoscopic images (Figures 7(b) and 7(c)) reconstructed by the SCANCO Medical system clearly show the bone-to-implant interface and trabecular microstructure around the implant.

BIC calculated by the SCANO evaluation software was in accordance with the change of trabeculae around the implant.  Figure 7(e) shows that BIC rate decreased from 4 weeks to 8 weeks in the diabetic group, making the difference of BIC rate significant among the 3 groups 8 weeks after surgery.

The undecalcificated bone slices (Figure 7(f)) show that all implants were osseointegrated to the surrounding bone to different extents in three groups, as partial or total direct contact was observed between the implant and the host bone. A newly formed thin bony layer was found to surround the implants in all groups 4 weeks after implantation. After 8 weeks, the bony layer became thicker and covered more area around implants in the control and diabetic-GL groups. However, less bony contact to the implant was observed in the diabetic group and this bony layer appeared thinner. As Figure 7(g) shows, the BIC rate of the control group was 0.774 ± 0.094 after 4 weeks, slightly higher than that of the diabetic group (0.681 ± 0.101, *P* > 0.05) and the diabetic-GL group (0.753 ± 0.101, *P* > 0.05). At the end of the 8th week, the difference became significant. The BIC rate reached 0.888 ± 0.075 in the control group but dropped to 0.555 ± 0.099 in the diabetic group. In the diabetic-GL group, the BIC rate was 0.735 ± 0.096, notably higher than that of the diabetic group (*P* < 0.01).  Figure 7(h) shows that from 4 weeks to 8 weeks, the BIC rate slightly increased in the control and diabetic-GL groups. While in the diabetic group, BIC rate dramatically decreased as time moved on (*P* < 0.05).

**Figure 8:**
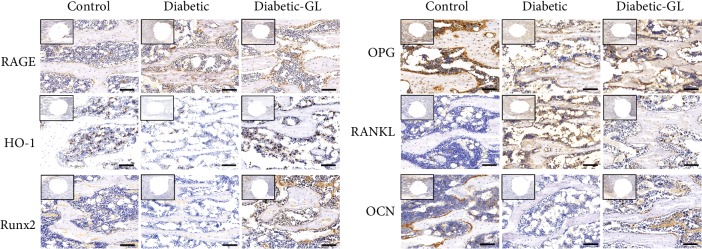
Immunochemistry evaluation of RAGE and HO-1 of the trabecular around the implant and effect of HMGB1 inhibiting on the expression of OPG, RANKL, Runx2, and OCN. The brown color indicates positive cells. Scale bar = 100 *μ*m.

IHC staining was performed to investigate the oxidative stress mediation and osteogenic differentiation in diabetic rat bone tissue under GL treatment. IHC results for HMGB1, RAGE, HO-1, RANKL, OPG, and Runx2, OCN expression (Figures 5(b) and 8) mirrored the gene expression results described above. Compared to normal rats, HMGB1 and RAGE were clearly upregulated in diabetic rats. While under GL treatment, the overexpressions of HMGB1 and RAGE were downregulated. In bone tissues of diabetic rats, the osteoblastic cells which lined the trabecular surface were intensely stained with HMGB1 or RAGE antibody. Moreover, many cells in the bone marrow were stained with HMGB1 or/and RAGE antibody, too. The control group and diabetic-GL groups showed significantly less stains in osteoblastic cells or bone marrow cells. More intense expression of the antioxidative HO-1 was observed around the implant of diabetic-GL rats than the diabetic rats. In addition, Runx2, OCN, OPG, and RANKL were evaluated. Increased expression level of bone formation molecules such as Runx2 and OCN and decreased bone resorption marker RANKL were demonstrated in the diabetic-GL group. Results indicate that inhibiting HMGB1 by GL can be effective to alleviate diabetic oxidative stress and in favor of bone formation.

## 4. Discussion

Bone-to-implant contact is impaired in patients with diabetes mellitus. However, the specific mechanism of poor osseointegration in diabetic condition remains to be further elucidated. Oxidative stress owing to diabetes mellitus has negative effects on bone quality and strength [22]. It has recently been suggested that HMGB1 participated to mediate cell injury caused by oxidative stress and inflammatory response [23] and interaction with overexpressed RAGE under diabetic condition [13]. Based on these evidences, we hypothesized that HMGB1 may play a critical role in the impaired implant osseointegration under diabetic environment.

HMGB1 was significantly overexpressed in both HG-treated BMSCs and peri-implant bone tissue and showed an excessive release in the plasm of diabetic rats. Cellular stress or inflammation stimulates the translocation of HMGB1 from nucleus to cytoplasm, induces culminates of HMGB1 cytoplasmic accumulation, and subsequently causes HMGB1 release into the extracellular milieu [24, 25]. The results were in accordance with a cross-sectional investigation in China, which revealed that plasma HMGB1 level increased in diabetic patients [10]. In addition, other studies have shown that HMGB1 is significantly elevated in the plasma of patients with other inflammatory or immune-related diseases. The plasma of HMGB1 in healthy people (and animals) was around 5 ng/ml (0.2 mM), while in patients with sepsis, serum HMGB1 reached 150 ng/ml (6 mM) [26]. Reported values of HMGB1 levels in synovial fluid from patients with rheumatoid arthritis ranged from 50 ng/ml to 10.4 mg/ml [27, 28].

GL is produced by the licorice plant *Glycyrrhiza glabra*. Our study reported that GL was unable to reduce blood glucose in diabetic rat. On the contrary, some studies demonstrated that GL could ameliorate hyperglycemia under diabetic condition. Liu et al. [29] reported that GL treatment (drinking water, 150 mg/kg/day) reduced blood glucose levels of diabetic mice and lasted for about two months. In Sen et al.'s study [30], blood glucose level in diabetic rats treated with GL (100 mg/kg, intraperitoneal injection) returned to almost normal levels two weeks after a single administration. Considering the more than 100 diabetic rats in our experiment and the preliminary experiment we conducted using the same dose of GL (100 mg/kg, intraperitoneal injection, data not shown), we are skeptical about Sen's result. Our data do agree with Abu El-Asrar et al.'s study [31], which reported no differences in blood glucose level at one month of GL treatment (drinking water, 150 mg/kg/day) in diabetic rats. The variations in experimental animal species, disease models, dosages, and administration methods may be partly responsible for the differences. However, the exact reason remains unclear.

GL acts as a direct inhibitor of HMGB1. Under normal glucose, the effect of GL on HMGB 1 is modest (data not shown). However, under high-glucose condition, where HMGB1 is overproduced and released, GL can inhibit the production of HMGB1 and its binding to the receptor after release. In this study, it is reasonable to verify a significant decrease of the gene expression of HMGB1 and HMGB1-RAGE binding in BMSCs treated with GL (10 *μ*M) and rats administrated with GL (50 mg/kg). In published papers, GL was reported to downregulate HMGB1 expression in lung cancer cells [32] and in rat brain tissue after acute subarachnoid hemorrhage [33]. Moreover, GL can bind directly to HMGB1, and potentially block the HMGB1 released into the extracellular space and inhibited its extracellular activities [34].

Otherwise, increased expression of RAGE was observed in HG-treated BMSCs and diabetic bone tissue, while inhibition of HMGB1 by GL caused a corresponding decreased expression of RAGE. The increase of RAGE level under HG condition may be due to the increased RAGE feedback expression in response to the accumulation of RAGE ligands such as HMGB1, AGEs, and inflammatory mediators. The expression of RAGE in BMSCs appears to be upregulated by its ligand stimulation. It has been reported that accumulation of AGEs can induce RAGE expression in MSCs and osteoblasts, which is closely related to bone loss [35]. The HMGB1 inhibitor GL has no direct effect on RAGE, and the reduction of RAGE level by GL treatment may be related to its inhibition of HMGB1 release and binding to HMGB1, which resulted in decreased RAGE ligand levels. GL is bound to HMGB1 in a concentration-dependent manner and the equilibrium dissociation constant (Kd) was 4.03 *μ*M. Moreover, HMGB1-RAGE binding was inhibited by GL, with the half maximal inhibitory concentration (IC50) being 6 *μ*M [13]. GL interacts with the A- and B-box of HMGB1 molecule [36], and the modification of HMGB1 can strongly interfere its binding with RAGE [37]. Thus, HMGB1 binding to GL inhibits its binding to RAGE, resulting in the reduction of RAGE signaling.

Cell proliferation and differentiation are two processes directly correlated to bone regeneration. Therefore, effects of HMGB1 hampering proliferation and differentiation of osteoblastic BMSCs attenuate bone healing and implant integration to the surrounding bone. With elevated HMGB1 expression, cell proliferation and differentiation were downregulated in HG-treated BMSCs, showing as inhibited proliferation, diminished ALP activity, impaired calcium deposition *in vitro*, and reduced BIC *in vivo*. Moreover, released HMGB1 upregulated the expression of RANKL in BMSCs and in bone tissue. RANKL binds to RANK receptor, a member of the TNF receptor family locating to BMSCs and osteoclasts, and promotes osteoclast differentiation and activation. OPG can attenuate this action, as it is a decoy receptor for RANKL. The increase of RANKL/OPG ratio may contribute to the differentiation of osteoclasts which can lead to bone resorption [38]. Zhou injected HMGB1 antibody together with RANKL into mice and observed that RANKL-induced osteoclastogenesis was weakened [39], suggesting that extracellular HMGB1 played an important role in RANKL-induced osteoclastogenesis.

Moreover, studies have shown that in osteoclast precursor cells, RAGE appears to be required for integrin signaling and the cross-talk between HMGB1 and integrin signaling [39]. RAGE regulates integrin signaling at the transcriptional level, on the cell surface and intracellularly [40]. Osteoclasts from RAGE mutant mice showed disrupted actin loop structure and defective integrin signaling [40], which is required for osteoclast activation. Thus, RAGE regulating integrin signaling and actin loop formation may be the basis for HMGB1-induced osteoclastogenesis. Blockage of HMGB1 can be an alternative way to protect bone from resorption as it can partly rescue the increased RANKL/OPG ratio. However, unlike our research, Martinotti et al. found that HMGB1 promoted osteoblastic SaOS-2 cell proliferation, migration, and osteogenic differentiation at the concentration of 2.5 nM [41]. We propose that acting as an essential nuclear protein and an important inflammatory mediator, a modest amount of HMGB1 can regulate the number and behavior of osteoblasts and osteoclasts to be recruited to the injury site for bone regeneration. While under pathological conditions, such as diabetic oxidative stress, periodontitis, and arthritis, an uncontrolled expression and release of HMGB1 can lead to an overwhelming of bone resorption.

The change of trabecular characters and BIC rate suggests that with the prolongation of time, new bone formation around the implant took the lead in the control group, while bone resorption dominated in the diabetic group. The significantly increased RANKL/OPG ratio may account for the decreased bone volume and deteriorated osseointegration in diabetic rats in the second 4-week phase. After inhibiting HMGB1 with GL, the trabecular number, thickness, bone volume, and BIC rate were significantly increased compared with the diabetic group, indicating that HMGB1 may play a role in bone resorption process. Therefore, it can be inferred that inhibiting HMGB1 achieves to improve trabecular structure and osseointegration in diabetic rats by depressing diabetic bone resorption. This is consistent with the results that inhibiting HMGB1 can significantly reduce RANKL/OPG in BMSCs and inhibit osteoclast differentiation. According to Wolf et al.'s research, HMGB1 shows a RANKL-related activity in regulating periodontal remodeling during orthodontic tooth movement in rats [42]. Yoshihara-Hirata et al. found that HMGB1 was involved in the development of periodontitis and anti-HMGB1 antibody treatment attenuated alveolar bone absorption and the progression of periodontitis [43]. These results provide strong support for this study. However, more research is required to elucidate the role of HMGB1 in osteoclastogenesis, as well as its form of participation, either alone or in cooperation with other osteoclast-inducing signaling molecules.

According to our results, increased expression of HMGB1 and the HMGB1-RAGE interaction can be implicated in diabetic osseointegration impairment. Previous researches have demonstrated the pathological roles for HMGB1-RAGE interaction involved in diabetic nephropathy [44] and diabetic retinopathy [45]. These findings support that HMGB1 interacting with RAGE plays a significant role in diabetes-related complications. Considering the relationship among HMGB1-RAGE, diabetes, and oxidative stress, we focused on the role of ROS. Sustained increased oxidative stress and associated mediators play an important role in most chronic human diseases, including diabetes [46]. In this study, we observed an increased ROS accumulation in HG-treated BMSCs, increased lipid peroxidation products (MDA), and decreased free radical scavenger (GSH-PX) in diabetic rats. An increased SOD activity in diabetic rats was observed in diabetic rats at the first 4 weeks, which could be partly accounted by a compensatory mechanism in response to oxidative stress overload. By 8 weeks, SOD activity decreased significantly in the diabetic rats. The long-lasting increase of free radical in diabetes may cause this depletion in SOD activity. Inhibiting HMGB1 showed obvious signs of alleviating oxidative stress by the protection of ROS buffering mediators in comparison with the diabetic group. Results from western blot and immunohistochemistry showed that the expression of HO-1 was decreased in BMSCs and the bone tissue under HG or diabetic condition and was increased after inhibiting HMGB1, suggesting that inhibition of HMGB1 can upregulate the antioxidant protein HO-1, therefore alleviating oxidative stress damage in BMSCs and bone tissue.

Under diabetic condition, cellular oxidative stress or inflammation stimulates the transport of HMGB1 from the nucleus to the cytoplasm, resulting in the accumulation of HMGB1 in the cytoplasm and subsequent release into the extracellular environment [24, 25]. When secreted or released into the extracellular environment, HMGB1 acts as a powerful mediator of metabolic activity and inflammation, depending on the location of HMGB1, its molecular binding ligands, and cellular redox status [47]. Studies have found that the redox state of HMGB1 is the main factor in determining its immune activity [48–51]. These authors suggest that altering the oxidative stress environment can reduce inflammation and tissue damage by modulating HMGB1 function. Depending on its structure, HMGB1 may or may not induce cytokine production and play different roles. Therefore, on the one hand, HMGB1 interact with RAGE, leading to ROS overproduction and accumulation. The decrease in HMGB1-RAGE binding was accompanied by a decrease in ROS accumulation. On the other hand, oxidative stress can stimulate the release of HMGB1 from nucleus towards cytoplasm and regulates its function [52]. Moreover, redox also regulates the expression and function pattern recognition receptors such as RAGE [53]. Therefore, a vicious circle of HMGB1-RAGE and ROS begins.

## 5. Conclusions

In conclusion, our results provide evidences that HMGB1 is a pathological cytokine relevant to diabetic impairment of osseointegration, and glycyrrhizin possesses protective activity on HMGB1/RAGE-induced BMSC dysfunction and diabetic oxidative stress, providing novel strategies to achieve improved osseointegration in diabetic patients.

## Figures and Tables

**Table 1:** Primer sequences for real-time qPCR analysis of the mRNA expression.

Gene	Primers (5′-3′)	
HMGB1	F: GGCTTATCCATTGGTGATGTTGC	R: TTCTCATACTTCTCCTTCAGCTTGG
RAGE	F: CAACTACCGAGTCCGAGTCTACCA	R: AGAGGTTTCCCATCCAAGTGC
HO-1	F: ACCTTCCCGAGCATCGACAA	R: TCTTAGCCTCTTCTGTCACCCTGT
OPG	F: TTGGCTGAGTGTTCTGGTGGA	R: CTGGAAAGTTTGCTCTTGCGA
RANKL	F: TCGGGTTCCCATAAAGTCAGTC	R: CAAATGTTGGCGTACAGGTAATAGA
ALP	F: AACCTGACTGACCCTTCCCTCT	R: TCAATCCTGCCTCCTTCCACTA
Runx2	F: CGGGAACCAAGAAGGCACA	R: GCGGGACACCTACTCTCATACTG
OCN	F: GTGCAAAGCCCAGCGACTCT	R: GCTCCAAGTCCATTGTTGAGGTAG
*β*-Actin	F: TGACGTTGACATCCGTAAAGACC	R: TGCTAGGAGCCAGGGCAGTAA

F: forward; R: reverse.

## Data Availability

The data used to support the findings of this study are available from the corresponding author upon request.
